# MRI-based volume measurement methods for staging primary lower extremity lymphedema: a single-center study of asymmetric volume difference-a diagnostic study

**DOI:** 10.1186/s12891-023-06912-x

**Published:** 2023-10-12

**Authors:** Mengke Liu, Yan Zhang, Xingpeng Li, Qi Hao, Bin Li, Rengui Wang

**Affiliations:** 1 Department of Radiology, Affiliated Beijing Shijitan Hospital, Capital Medical University, Beijing, 100038 China; 2 Department of Radiology, Affiliated the ninth Clinical Medical College, Peking University, Beijing, 100038 China; 3 Department of MRI, Affiliated Beijing Shijitan Hospital, Capital Medical University, Beijing, 100038 China

**Keywords:** Lymphedema, Magnetic resonance imaging, Lower extremity

## Abstract

**Background:**

Lower extremity lymphedema (LEL) staging is mainly assessed by systems that solely depend on physical examinations and lack quantitative assessment based on modern imaging.

**Objective:**

To explore the value of MRI-based asymmetric volume measurements in the clinical staging of primary LEL.

**Methods:**

92 patients with unilateral primary LEL underwent MRI examinations to determine the volume of the mid-calf (Vcl) calculated using the clinical dermatome method as well as the total volume (Vmri), musculoskeletal volume (VM), and subcutaneous volume (VS) volume of the middle calves. The difference between Vmri (DVmri) and VS (DVS) of the affected and unaffected calves was obtained and defined as the asymmetric volume difference. Meanwhile, the volume of the mid-calf (Vcl) and the difference in volume (DVcl) were calculated using the clinical circumferential method. The relationship between the asymmetric volume difference and clinical staging was then evaluated. Interobserver consistency was assessed through the intraclass correlation coefficient (ICC). Volume comparisons between the three groups were performed using the one-way analysis of variance (ANOVA) or the Kruskal-Wallis test. Spearman’s correlation was used to assess volume and clinical stage correlation. The receiver operating characteristic (ROC) curve was used to evaluate the value of asymmetric volume difference for clinical staging.

**Results:**

The asymmetric volume difference was statistically significant in stage I compared to stages II and III (*p* < 0.05). The asymmetric volume difference (DVmri: r = 0.753; DVS: r = 0.759) correlated more with the clinical stage than the affected Vcl (r = 0.581), Vmri (r = 0.628), VS (r = 0.743), and DVcl (r = 0.718). The area under the ROC curve (AUC) for identifying the clinical stage by the asymmetric volume difference was greater than that for the affected Vcl, Vmri, VS, and DVcl, with DVS (AUC = 0.951) having the largest area under the curve to distinguish between stages I and II.

**Conclusion:**

MRI-based asymmetric volume difference is an adjunctive measure for LEL clinical staging with good reproducibility. DVS could be the best indicator for differentiating between stages I and II of primary LEL.

## Introduction

Lower extremity lymphedema (LEL) is a relatively common condition often associated with late diagnoses and is characterized by abnormal distribution and infiltration of lymphatic fluid in subcutaneous soft tissues, abnormal proliferation of fibers and fat, and susceptibility to infection [1]. The International Society of Lymphology (ISL) classifies primary or secondary LEL depending on the cause [2]. Primary LEL is rare, with a prevalence of about 1 in 100,000, occurs more frequently in women than men, and usually occurs in childhood. Primary LEL is caused by several genetic mutations that result in lymphatic duct hypoplasia and restricted lymphatic drainage [3].

The International Society of Lymphology (ISL) classifies LEL into stages 0, I, II, and III based on the clinical presentation of the patient [2]. Stage 0 generally presents with insignificant symptoms. Stage I is marked by protein-rich fluid accumulation in the interstitium, which causes a mild depression of the limb when pressed; the degree of swelling can be reduced by elevating the limb ^2^. In stage II, there is obvious depression in the pressed limb, and due to fat hypertrophy and fibrous connective tissue hyperplasia under the stimulation of inflammatory cytokines, the degree of swelling cannot be reduced entirely after elevating the limb [2]. In stage III, the skin and subcutaneous tissue fibrosis is further developed. While no obvious depression of the limb when pressed is observed, skin pigmentation, acanthosis, warty hyperplasia, and even elephantiasis would appear [2]. Currently, the staging of lymphedema is based only on symptoms and signs at the time of the patient’s examination and lacks quantitative assessment based on imaging [3]. Lymphedema is a dynamic process of change, and from the point of view of clinical examination, the progression of the patient’s process is slow, not easily detectable, and hence is often overlooked by clinicians. Therefore, finding a method that allows a more objective and quantitative assessment of the severity of lymphoedema in patients with primary LEL and monitoring its progression could provide the most direct and transparent reference for patients in terms of calculating the most concrete value in understanding the progression of lymphoedema, developing an appropriate treatment plan, and understanding the effectiveness of treatment.

Lymphoscintigraphy has been the standard method for LEL diagnosis as it can visualize the lymphatic vessels and nodes in the limbs, the continuity and fluidity of lymphatic flow, the presence of collateral lymphatic vessels and lymphatic fluid subcutaneous reflux, lymphatic leakage, and abnormalities of the lymphatic system in other parts besides the limbs [4]. However, lymphoscintigraphy has low spatial resolution and struggles to visualize fine anatomical structures, which makes it difficult to assess the stage of limb swelling in patients with lymphedema [5]. An alternative method for measuring limb volume is magnetic resonance imaging (MRI), which is non-invasive, non-radioactive. It has a much higher soft-tissue resolution than lymphoscintigraphy, with a more extensive scanning range than ultrasound [6–8]. MRI has been increasingly used to evaluate primary LEL because it can discern the distribution of lymphatic fluid within the subcutaneous soft tissues of the affected limb [6], making MRI an ideal modality for assessing the stage of lymphedema.

In this study, we present a simple, reproducible, and accurate MRI-based measurement defined as the limb asymmetry volume difference to assess the clinical staging of primary LEL. Then, we compared this method to the clinical circumference method. We aimed to explore the clinical value of MRI-based asymmetric volume measurements in the clinical staging of primary LEL.

## Methods

### Study sample and patients

The institutional review board approved this retrospective study of opportunistic screening, and the requirement to obtain informed consent was waived.

The cohort of this study was identified by searching the institutional picture archiving and communication systems (PACS) database for medical records from January 2017 to January 2020 to identify patients with primary unilateral LEL. The inclusion criteria included: (1) lymphoscintigraphy diagnosis of unilateral lower extremity lymphedema, (2) no treatment prior to hospital admission, (3) swelling of the lower extremities with no apparent cause. The exclusion criteria included: (1) lymphoscintigraphy diagnosis of bilateral lower extremity lymphedema; (2) prior submission to surgical treatments; (3) secondary factors such as surgery for a malignant tumor, trauma, filariasis, and infection. The final cohort consisted of 92 patients (56 females, 36 males; age range: 10–69 years; median age: 28.5 years). Referring to the 2020 ISL clinical staging criteria [2] (Table 1), the patients were classified as stages I, II, or III primary LEL, with no stage 0 patients in this study.

**Table 1:** Clinical (According to the 2020 Consensus Document of the International Society of Lymphology) Characteristics of Lymphoedema Stages

	Clinical characteristics
Stage 0	Latent or subclinical condition. Swelling is not present despite impaired lymph transport.
Stage I	Early accumulation of fluid relatively high in protein content which decreases with limb elevation. Pitting may occur.
Stage II	Limb elevation alone rarely decreases the tissue enlargement. Pitting is present.
Stage III	Lymphostatic elephantiasis; pitting can be absent; trophic skin changes such as acanthosis, abnormalities in skin characteristics and thickness, fat and fibrosis deposition, and warty overgrowths are present.

### Clinical circumferential method lateral volume

A clinically experienced lymphatic surgeon measured the bilateral circumference of the patient’s lower extremities and the circumference at the level of the upper 1/3 (C1) and lower 1/3 (C2) of the calf on the affected and healthy sides, respectively. The distance between these two levels (H) was recorded, and volume V was derived using the truncated cone formula (1). The volume between the upper and lower 1/3 of the calf was defined as the mid-calf volume (Vcl), and the difference in volume (DVcl) of the bilateral calves was calculated.

1 $$v=\frac{(C1\times C1+C1\times C2+C2\times C2)H}{12\pi }$$

### MR imaging and measurement methods

All MRI examinations were performed on a 1.5T scanner (Intera Ingenia, Phillips, Best, the Netherlands) equipped with an eight-channel torso array coil, using a Short Term Inversion Recovery (STIR) sequence. The STIR parameters were TR 8549 ms, TE 165 ms, FOV 400 mm×400 mm, slice thickness 7 mm, and slice spacing 0.4 mm.

Post-processed images were reviewed by two independent readers (ML and BL with 10 and 20 years of experience in musculoskeletal MRI, respectively) blinded to the clinical stage. Measurements of the soft tissue area of the calf were performed on a PACS workstation (ATLASPro1818.2021). STIR images were selected for area measurements to eliminate the influence of fatty tissues within the subcutaneous soft tissues on the measurement results.

The total area and musculoskeletal area, in cm^2^, were measured at the upper (S1) and lower (S2) 1/3 levels of the calf, respectively. The upper 1/3 of the calf was defined as the image plane corresponding to the upper 1/3 of the line from the tibial plateau to the level of the external ankle. The lower 1/3 of the calf was defined as the image plane corresponding to the lower 1/3 of the line from the tibial plateau to the level of the external ankle.

**Fig. 1:**
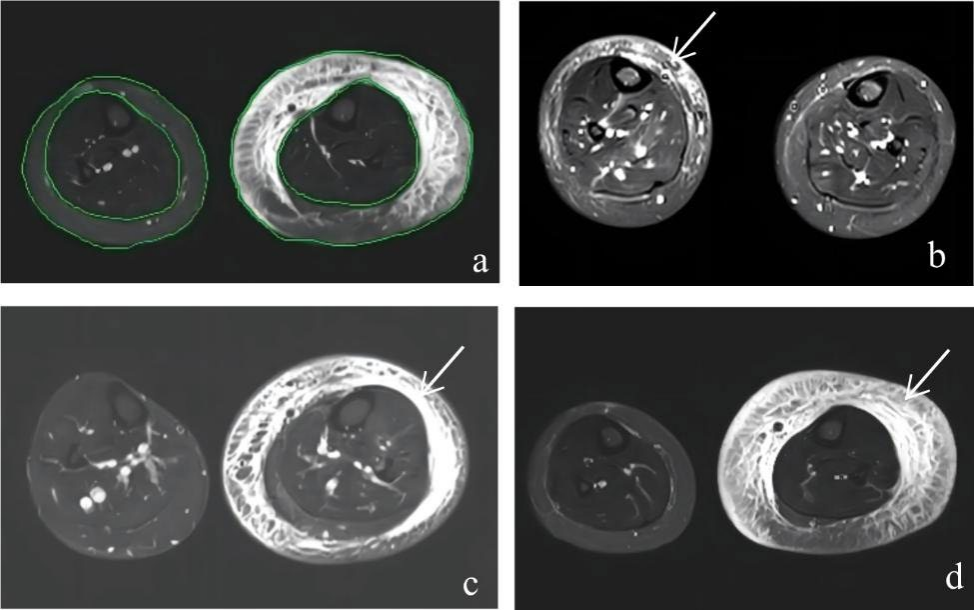
**(a)** MRI measurement of soft tissue in the calf: the boundary between subcutaneous soft tissue and muscle in the cross section is the superficial fascia, the area enclosed by the skin is the total area, and the area enclosed by the superficial fascia is the bony muscle area. **(b**–**d)** Cross-sectional STIR images of the lower limb showing soft tissue changes in different clinical stages of lymphedema: **(b)** primary lower limb lymphedema of stage I (10-year-old female, right calf); **(c)** primary lower limb lymphedema of stage II (44-year-old male, left calf); **(d)** primary lower limb lymphedema of stage III (29-year-old male, left calf). Schematic representation of the lymphedema area (arrow)

By manually drawing the border of the skin edge and the superficial fascia between the muscle and the subcutaneous soft tissue with a mouse, the workstation could automatically calculate the total area and the bony muscle area, as revealed in Fig. 1. The distance between the two planes (H) was measured and recorded. The total volume (Vmri) and the musculoskeletal volume (VM) between the upper 1/3 and lower 1/3 of the bilateral calves were calculated using the circular table volume formula (2). The subcutaneous soft tissue volume (VS) is the difference between the total and musculoskeletal volumes. The bilateral calves’ total volume (DVmri) and subcutaneous soft tissue volume (DVS) were then calculated as the asymmetric volume difference.

2 $$V=\frac{(S1+S2+\sqrt{(S1\times S2)})H}{3}$$

### Statistical analysis

Data conforming to the normal distribution are expressed as mean ± standard deviation; otherwise, they are expressed as median ± interquartile spacing. Statistical analysis was performed using SPSS version 23.0 (SPSS, Inc., Chicago, IL, USA). A Kolmogorov-Smirnov (KS) test was adopted to determine whether the data conforms to a normal distribution. The independent sample t-test (for normal distribution) or Mann-Whitney U test (for non-normal distribution) was used to compare the affected and unaffected soft tissue volumes. The Kruskal-Wallis testing (for non-normal distribution) or one-way analysis of variance (ANOVA; for normal distribution) was used to compare the three stages (LEL stages I, II, and III), followed by Bonferroni’s post-hoc test for comparing between groups at a significance level of *p* < 0.05. Spearman’s correlation analysis was used to assess volume and clinical stage correlation. *p* < 0.05 was considered a statistically significant difference.

The receiver operating characteristic (ROC) curve was drawn using MedCalc 19.1 (MedCalc Software bv, Ostend, Belgium; https://www.medcalc.org; 2019) to assess the diagnostic value of volume for clinical staging and to determine cut-off values, sensitivity, and specificity. *p* < 0.05 was considered a statistically significant difference.

## Results

### Interobserver consistency

There was excellent interobserver agreement between both readers regarding measurements of Vmri in the affected (ICC = 0.999) and contralateral (ICC = 0.991) calves and VM in the affected (ICC = 0.995) and contralateral (ICC = 0.985) calves. The mean measurement value of two readers was used as the final result.

### Consistency of clinical and MR measurements of mid-calf volume

The mean volume of the affected Vcl was 1454.33 ± 518.63 cm³, and that of the affected Vmri was 2188 ± 398 cm³; the difference was not statistically significant (*p* > 0.05). The mean volume of the unaffected Vcl was 955.85 ± 215.85 cm³, and that of the unaffected Vmri was 911.10 ± 212.39 cm³; the difference was not statistically significant (*p* > 0.05). There was an excellent inter-observer agreement between V_Cl_ and V for the affected limb (ICC = 0.945) and the healthy limb (ICC = 0.901).

### Comparison of soft tissue volumes in the calf measured bilaterally via MRI

Vmri and VS were significantly greater on the affected side than on the unaffected one (*p* < 0.05). In contrast, the VM of the affected and unaffected limbs were not significantly different (*p* > 0.05, Table 2). Correlation analysis showed a strong correlation between Vmri and VS (r = 0.927) and a low correlation between Vmri and VM (r = 0.466) on the affected side (*p* < 0.05). At the same time, there was a significant positive correlation between Vmri and VM (r = 0.874) as well as VS (r = 0.781) on the unaffected side (*p* < 0.05), especially with VM, and the correlation was more significant than that with VS.

**Table 2:** Soft-tissue volume of affected versus unaffected lower extremity

	Affected (n = 92)	unaffected (n = 92)	*T/Z*	*p*
Vmri	1300.35±616.62^1^	911.11±212.39	8.479	< 0.001^†^
VM	630.46± 148.66	631.91±144.29	−0.067	0.947
VS	704.01±585.10^1^	252.48±170.05^1^	10.342	< 0.001^†^

Vmri: total volume of the mid-calf;VM: osteomyotomus volum of mid-calfe; VS: subcutaneous soft tissue volume in the mid-calf

^1^: median ± interquartile range; ^†^: Mann-Whitney U Test

### Assessment of the clinical staging of LEL by the volume of the affected mid-calf

The correlation between VS of the affected calf (r = 0.743) and the clinical stage was higher than that of Vmri (r = 0.628), Vcl (r = 0.581), and DVcl (r = 0.718). Vcl, DVcl, Vmri, and VS increased proportionally with the LEL clinical stage, and ANOVA within the different clinical stages was significantly different (*p* < 0.05). When comparing the two groups, Vcl, DVcl, Vmri, and VS values of stage I patients were significantly smaller than those of stage II and stage III patients (*p* < 0.05). In contrast, the difference between stages II and III was not statistically significant (*p* > 0.05, Table 3).

**Table 3:** The soft-tissue volume of the affected and unaffected calf corresponding to stage of lower extremity lymphoedema

	I (n = 33)	II (n = 40)	III (n = 19)
Vcl			
affected	1122.01 ± 249.02^ab^	1599.04 ± 421.35	1915.01 ± 673.07^b^
unaffected	967.48 ± 184.48	934.47 ± 191.42	980.65 ± 306.18
DVcl	154.52 ± 143.91^ab^	664.57 ± 352.77	934.36 ± 600.52^b^
Vmri			
affected	1086.28 ± 217.70^ab^	1535.89 ± 410.26	1921.90 ± 645.79^b^
unaffected	925.27 ± 166.13	866.05 ± 200.61	939.26 ± 298.64
DV†	161.01 ± 120.29^ab^	649.84 ± 335.13	851.46 ± 529.50^1b^
VS			
affected†	457.63 ± 142.53^ab^	906.84 ± 338.01	1088.95 ± 837.80^1b^
unaffected	292.35 ± 95.81	259.93 ± 96.11	296.90 ± 153.93
DVS†	165.27 ± 118.35^ab^	646.90 ± 310.12	779.74 ± 614.60^1b^

Vcl: total volume of the mid-calf by clinical; DVcl, difference of Vcl; Vmri: total volume of the mid-calf; VS: subcutaneous soft tissue volume in the mid-calf; DVmri, difference of total volume; DVS, difference of subcutaneous soft tissue volume; ^1^: median ± interquartile range; ^†^: Kruskal-Wallis Test; ^a^, the *p*-value for statistical comparisons between stage I and stage II was less than 0.01; ^b^, the *p*-value for statistical comparisons between stage I and stage III was less than 0.01

**Fig. 2:**
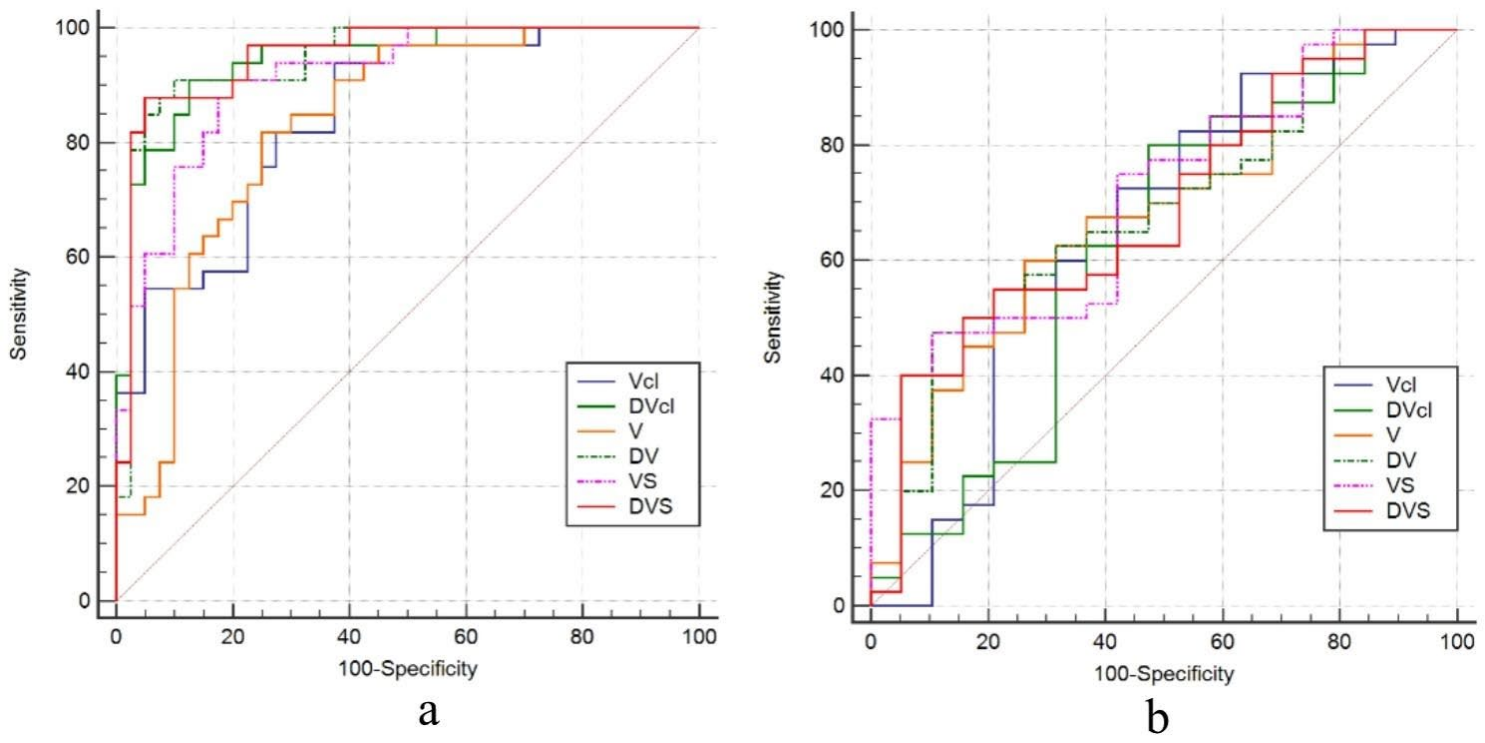
ROC curves showing the efficacy of Vcl, V, VS of the affected calf and the difference with the corresponding measurements on the healthy side (DVcl, DV, DVS, respectively) for clinical staging of primary lower limb lymphedema. **(a)** Stage I compared with stage II; **(b)** stage II compared with stage III.

ROC curve analysis showed that the mid-calf volume was significantly more effective in discriminating lymphedema stage I from stage II than stage II from stage III. The area under ROC curve (AUC) was higher for VS (AUC = 0.912) than for Vmri (AUC = 0.831) and Vcl (AUC = 0.847) in discriminating patients with stages I and stage II lymphedema (Fig. 2). The AUC values for DVcl (AUC = 0.945) were higher than VS. The AUC, cut-off values, sensitivity, and specificity of Vcl, DVcl, Vmri, and VS for identifying different clinical stages of primary LEL are listed in Tables 4 and 5.

**Table 4:** ROC analysis of soft-tissue volume to identify primary lower extremity lymphedema stage I and II

	AUC	*p*	95%CI	Cut-off value	sensibility	specificity
Vcl	0.847	0.001	0.744 to 0.921	1418.33	93.94	62.50
DVcl	0.945	0.001	0.865 to 0.985	330.60	90.91	87.50
Vmri	0.831	0.001	0.725 to 0.909	1206.19	81.82	75.00
DVmri	0.945	0.001	0.866 to 0.985	286.76	90.91	90.00
VS	0.912	0.001	0.822 to 0.966	618.70	90.91	80.00
DVS	0.951	0.001	0.873 to 0.988	278.48	87.88	95.00

AUC: Area under the ROC curve; CI: Confidence interval; Vcl: total volume of the mid-calf by clinical; Vmri: total volume of the mid-calf by MRI; VS: subcutaneous soft tissue volume in the mid-calf; DVcl, difference of Vcl; DVmri, difference of Vmri; DVS, difference of subcutaneous soft tissue volume

**Table 5:** ROC analysis of soft-tissue volume to identify primary lower extremity lymphedema stage II and III

	AUC	P	95%CI	Cut-off value	sensibility	specificity
Vcl	0.657	0.067	0.522 to 0.775	1580.46	55.00	78.95
DVcl	0.624	0.150	0.488 to 0.747	917.22	80.00	52.63
Vmri	0.683	0.014	0.549 to 0.798	1564.83	60.00	73.68
DVmri	0.679	0.017	0.579 to 0.822	542.10	47.50	89.47
VS	0.712	0.002	0.545 to 0.795	796.82	47.50	89.47
DVS	0.684	0.006	0.550–0.799	568.35	50.00	92.31

AUC: Area under the ROC curve; CI: Confidence interval; Vcl: total volume of the mid-calf by clinical; Vmri: total volume of the mid-calf by MRI; VS: subcutaneous soft tissue volume in the mid-calf; DVcl, difference of Vcl; DVmri, difference of Vmri; DVS, difference of subcutaneous soft tissue volume

### Assessment of lower extremity asymmetric volume difference for clinical staging

The correlation between DVmri (r = 0.753) and DVS (r = 0.759) and the clinical stage was significantly higher than Vmri, VS, Vcl, and DVcl on the affected calf, with DVS having the highest correlation with the clinical stage. DVmri and DVS increased gradually with the LEL clinical stage, and ANOVA within different clinical stage groups was significantly different (*p* < 0.001). The values of DVmri and DVS in stage I were significantly smaller than the values in stage II and stage III (*p* < 0.001), and the values of DVS and DVmri were not significantly different when compared between stages II and III (*p* = 0.333, 0.345, Table 3).

ROC curves showed that the AUC was 0.951 and 0.945 for DVS and DVmri to identify patients with stage I and stage II lymphedema, respectively, and the AUC was the highest for DVS to identify patients with stage I and stage II lymphedema with a 95% confidence interval (95%CI) of (0.873–0.988). DVS value of sensitivity (87.88%) and specificity (95.00%) was the highest at 278.48 cm³, which could be used as a cut-off value to distinguish stage I from stage II of primary LEL(Fig. 2). The AUC, cut-off values, sensitivity, and specificity of DVmri and DVS for identifying different clinical stages of primary LEL are displayed in Tables 4 and 5.

## Discussion

Our study found that the MRI-based volume measurement method has perfect repeatability. Furthermore, the asymmetric volume difference of the lower extremities calculated by MRI was sensitive to changes in the degree of limb swelling in LEL, which could quantitatively assess the stage of unilateral primary LEL and thus identify stages I and II of primary LEL. This procedure is also proven reproducible in the present study, as indicated by the consistent interobserver agreements achieved after repetitive measurements.

LI [9] found that the subcutaneous soft tissue in the mid-calf is especially prone to developing lymphedema and proposed a specific LEL diagnosis index to be developed based on the edema at the mid-calf level. Given the information above, this study selected the mid-calf volume as the region of interest (ROI).

MRI does not use radioactivity and has a high soft tissue resolution, allowing it to clearly distinguish the different tissues in the limb and better track the subtle progression of the swelling in early-stage primary LEL [10–12]. LEL is a chronic irreversible disease, and patients often need regular follow-up observations; therefore, the low cost of MR and its general acceptability among the general public in China encourages the patients to comply with follow-up routine and, in turn, improve the tracking of treatment effect and prognosis. In addition, the time required to scan a patient is approximately 6–7 min, and the time required to measure the mid-calf volume of a patient is approximately less than 1 min. Therefore, the time taken for an MRI scan and image measurement is considerably less than a standardized examination based on clinical staging criteria.

Clinical measurements cannot clarify the location of lesion occurrence within the limb. However, MR imaging can selectively measure the volume of different tissues in the limb and explore the relationship between each tissue and the swelling in the limb. In this study, we found that Vmri of the affected calf showed a strong positive correlation with VS (r = 0.927), which is higher than VM (r = 0.466). The VM of the healthy calf (r = 0.874) was more correlated with Vmri than VS (r = 0.781), and Vmri and VS of the affected limb were greater than that of the healthy calf, while VM of the affected calf was not significantly different from that of the healthy side. Lymphedema is generally understood not to involve the muscular compartment. Yoo et al. [13] and Li et al. [9] measured the volume and area of the subcutaneous region and bilateral limb muscles in patients with LEL based on CT and MRI, respectively, and found that there was no statistically significant difference in the area and volume of the bilateral limb muscles, confirming that lymphedema rarely involves the subfascial muscles, which is consistent with the results of the present study.

In this study, we also found that VS and DVS correlate better with clinical staging than Vcl and DVcl, implying that the MR measurements of the subcutaneous volume of the affected limb and the difference between the affected and unaffected limbs allowed a more accurate assessment of the clinical staging of LEL.

In this study, the asymmetric volume difference (DVmri, DVS) was statistically different between the three stages of lymphedema (*p* < 0.05). The difference between the two indices was significant between stages I and II and between stages I and III (*p* < 0.05), but not between stages II and III (*p* > 0.05). In the early stage of primary LEL, stagnant lymphatic fluid exudes into the interstitial space. The limb gradually swells, fat and fibrous tissues begin to overgrow, the skin gradually becomes fibrotic, and hypertrophic adipocytes and tissue fibrosis gradually replace the protein-rich lymphatic fluid in the interstitial space. The limb thickens and hardens, eventually losing elasticity, and the swelling halts [14]. The AUC value of this difference for identifying primary LEL in stages I and II was significantly higher than for identifying stages II and III, suggesting that the asymmetric volume difference may be more effective in identifying early lymphedema.

Based on the results of the study, we believe that the asymmetric volume difference obtained from MR-based volumetric measurements is more useful for clinical staging of limb lymphedema given the following advantages: (1) MR imaging distinguishes between different tissues, such as the subcutaneous fat, muscle, and bone of the lower limbs, while not limited to just the volume of the whole lower limbs. (2) Factors associated with body size interfere in determining the staging and assessment of lymphedema. The asymmetric volume difference can reduce the uncertainty caused by different body sizes between individuals, enabling individualized advantages. (3) The pathophysiology of MR-based volumetric assessment of limb lymphedema is largely consistent [15, 16]. Lymphatic return is obstructed due to lymphatic vascular dysplasia, reduced number or reduced function, and stagnant lymphatic fluid continuously leaks into the tissue interstices. It is confined to the subcutaneous tissue’s superficial interstices without involving the fascia’s muscles. As the disease progresses, slowed lymphatic return stimulates adipogenesis and fat deposition, followed by fibroblast activation and connective tissue proliferation, which accumulate around the subcutaneous soft tissues and further increase subcutaneous tissue swelling in the limb.

Among the asymmetric volume differences, DVS had the highest ROC curve for identifying stages I and II. We believe that DVS is most suitable for assessing the clinical stage of primary LEL, perhaps because DVS represents the increased volume of the subcutaneous fat layer on the affected limb compared to the other limb, which is fundamentally related to the swelling mechanism. One might even go as far as claiming that DVS indirectly reflects the edema volume. In this investigation, the sensitivity and specificity were the highest at 93.18% and 87.88%, respectively, when DVS was 278.48 cm³. The DVS value can be used as the cut-off value to distinguish between primary LEL stage I and II. Some studies used MRI to measure the calf soft tissue thickness in patients with secondary LEL and found that the difference in subcutaneous soft tissue thickness of bilateral limbs had the most significant diagnostic value in identifying adjacent clinical stages. They used it as the most sensitive measure of clinical staging of primary lymphedema, especially in the early diagnosis of lymphedema [17, 18].

In this study, MRI-based asymmetric volume differences have several shortcomings. (1) The effect of this method is less effective in bilateral LEL. (2) In this study, only mid-calf volumes were calculated, not the whole lower extremity. Another study is in progress that measures the whole lower extremity asymmetric volume differences.

## Conclusion

MRI-based asymmetric volume differences can be used as an adjunctive measure for clinical staging of LEL with good reproducibility. DVS could be the best indicator for differentiating between stages I and II.

## Data Availability

The datasets used and/or analyzed during the current study are available from the corresponding author on reasonable request.
